# RNA sequencing reveals transcriptomic changes in tobacco (*Nicotiana tabacum*) following *NtCPS2* knockdown

**DOI:** 10.1186/s12864-021-07796-8

**Published:** 2021-06-23

**Authors:** Lingxiao He, Huabing Liu, Changhe Cheng, Min Xu, Lei He, Lihua Li, Jian Yao, Wenjun Zhang, Zhengguang Zhai, Qinzhan Luo, Jutao Sun, Tiezhao Yang, Shixiao Xu

**Affiliations:** 1 College of Tobacco Science, Henan Agricultural University, National Tobacco Cultivation & Physiology & Biochemistry Research Centre, Scientific Observation and Experiment Station of Henan, Ministry of Agriculture, Zhengzhou, 450002 China; 2 Technology Center, China Tobacco Zhejiang Industry Co, Ltd., Hangzhou, 310008 China; 3 China National Tobacco Corporation Henan company, Zhengzhou, 450002 Henan China; 4 Hunan Tobacco Corporation Changsha Company, Changsha, 410007 Hunan China; 5 Guangxi Zhuang Autonomous Region Tobacco Corporation Baise Company, Baise, 533000 Guangxi China

**Keywords:** CRISPR-Cas9, *Nicotiana tabacum*, RNASeq, Labdanoid diterpenes, *cis*-Abienol, Genome editing, Terpenoids

## Abstract

**Background:**

Amber-like compounds form in tobacco (*Nicotiana tabacum*) during leaf curing and impact aromatic quality. In particular, *cis*-abienol, a polycyclic labdane-related diterpenoid, is of research interest as a precursor of these compounds. Glandular trichome cells specifically express copalyl diphosphate synthase (*NtCPS2*) at high levels in tobacco, which, together with *NtABS*, are major regulators of *cis*-abienol biosynthesis in tobacco.

**Results:**

To identify the genes involved in the biosynthesis of *cis*-abienol in tobacco, we constructed transgenic tobacco lines based on an *NtCPS2* gene-knockdown model using CRISPR/Cas9 genome-editing technology to inhibit *NtCPS2* function in vitro. In mutant plants, *cis*-abienol and labdene diol contents decreased, whereas the gibberellin and abscisic acid (ABA) contents increased compared with those in wild-type tobacco plants. RNA sequencing analysis revealed the presence of 9514 differentially expressed genes (DEGs; 4279 upregulated, 5235 downregulated) when the leaves of wild-type and *NtCPS2*-knockdown tobacco plants were screened. Among these DEGs, the genes encoding *cis*-abienol synthase, ent-kaurene oxidase, auxin/ABA-related proteins, and transcription factors were found to be involved in various biological and physiochemical processes, including diterpenoid biosynthesis, plant hormone signal transduction, and plant-pathogen interactions.

**Conclusions:**

The present study provides insight into the unique transcriptome profile of *NtCPS2* knockdown tobacco, allowing for a better understanding of the biosynthesis of *cis*-abienol in tobacco.

**Supplementary Information:**

The online version contains supplementary material available at 10.1186/s12864-021-07796-8.

## Background

Aroma is an important attribute of tobacco (*Nicotiana tabacum* L.) leaves. It is an indicator of tobacco quality and is influenced by a variety of chemical components [1]. An important aromatic substance in tobacco leaf surface secretions is *cis*-abienol, which belongs to the labdanoid diterpenoid family [2, 3]. Previous studies have reported that *cis*-abienol plays an important role in determining the aromatic characteristics of tobacco, and it is an important precursor in the chemical synthesis of amber-like substances [4–6], which can affect aromatic quality. Furthermore, *cis*-abienol is involved in plant resistance to insects [7, 8] and diseases [9]. Therefore, it is important to explore the *cis*-abienol synthesis pathway in tobacco to better understand how to create disease-resistant tobacco varieties with high-quality or characteristic aromas upon flue curing.

The biosynthesis of *cis*-abienol in tobacco was initially reported to be controlled by a single gene, *Abl* [10, 11], which is located on chromosome A [12]. Subsequently, Vontimitta et al. [13] used 117 doubled haploid lines and simple sequence repeat molecular markers to locate the genes regulating *cis*-abienol and sucrose ester accumulation and found that both genes are located on chromosome A. The genetic distance between two genes is 8.5 cM, and a total of 17 pairs of markers can be found in the linkage group. Among them, PT10324 and *Abl* are completely separated. The markers beside *Abl* are PT55091 and PT61373, with distances of 2.02 and 0.6 cM, respectively [13]. Copalyl diphosphate synthase 2 (CPS2) from the angiosperm *Cistus creticus subsp. creticus* was first analysed through prokaryotic expression and dephosphorylation. Then, gas chromatography-mass spectrometry (GC-MS) analysis revealed that CPS2 catalyses the formation of 13(E)-labden-8-ol-15-diphosphate, implying that *CPS2* is involved in the biosynthesis of *cis*-abienol [14]. In gymnosperms, *cis-abienol synthase* (*ABS/KS*) contains both class I and class II functional domains, as shown by cloning and characterizing the gene from balsam fir (*Abies balsamea*) via transcriptome sequencing [15]. Sallaud et al. [16] cloned *NtCPS2* and *NtABS* from tobacco and showed that both genes are involved in the biosynthesis of *cis*-abienol, which involves two steps. First, CPS-like catalytic activity yields 8-hydroxy-copalyl diphosphate with a normal configuration, which can then be converted to *cis*-abienol by the *NtABS* product [16–18]. No other diterpenoid synthase has been reported to use 8-hydroxy-copalyl diphosphate as a substrate in dicotyledons to date. In addition, promoter analysis of *NtCPS2* showed that it could drive the expression of the *GUS* gene in glandular hairs [16, 19, 20]. The identification of *NtCPS2* and *NtABS* is of great significance for breeding high-quality tobacco and future microbial metabolic engineering. From this knowledge base, other diterpenoid-synthesising genes can be cloned and identified.

Among tobacco types, *cis*-abienol accumulates at different levels. It is mainly found in oriental and cigar tobacco but not in flue-cured tobacco, Burley tobacco, or Maryland tobacco [1, 16, 21]. To study the variation in *cis*-abienol content among different types of cultivated tobacco, 157 varieties of tobacco with or without *cis*-abienol were selected, and the expression levels of *NtCPS2* and *NtABS* were analysed [16]. *NtABS* cDNA sequences did not differ among tobacco varieties, but two distinct polymorphisms were found in *NtCPS2* cDNA: an 8-bp insertion at position 275 and a G-T transversion at position 292 of *NtCPS2.* Both of these result in a stop codon, which leads to early termination and shortening of the encoded peptide chain. Because the encoded protein loses its active site, it also loses its original function [16]. Thus, *NtCPS2* is key for *cis*-abienol biosynthesis. However, the mechanism by which the metabolic pathway of labdanoid diterpenoids is influenced by *NtCPS2* in tobacco and the effects of *NtCPS2* knockdown on other metabolic pathways are still unknown.

In this study, we used CRISPR/Cas9 gene-editing technology to knock down *NtCPS2*. The CRISPR/Cas9 *NtCPS2* expression vector was constructed from the high-aroma strain 8306 and transformed *NtCPS2*-knockdown plants were obtained. A high-throughput RNA sequencing (RNA-seq) technique was used to compare expression profiles between mutant and 8306 plants. Sequencing results were verified using fluorescence quantitative polymerase chain reaction (PCR), and physiological changes and transcriptional inheritance were analysed. By elucidating the function of the *NtCPS2* gene and the molecular mechanisms underlying the influence of its related genes, high-aroma tobacco varieties can be cultivated.

## Results

### Targeted mutagenesis of *NtCPS2* by CRISPR/Cas9 in tobacco

**Fig. 1:**
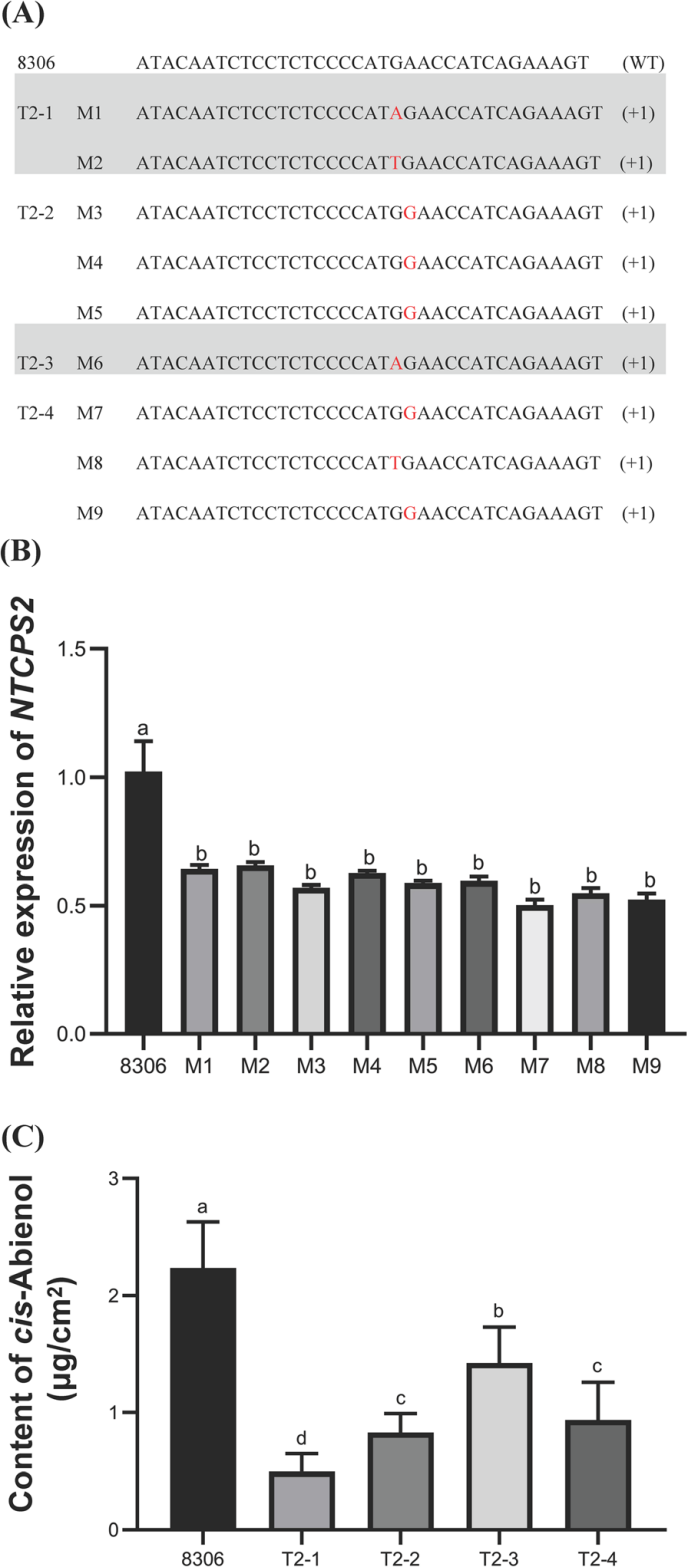
CRISPR/Cas9-induced mutations in T2 transgenic tobacco plants. **A** Sequences reflecting targeted inversions in the mutants. **B** Relative expression of *NTCPS2* in the mutants. **C** Quantification of *cis*-abienol in homozygous T2 transgenic tobacco plants. Values are averages of at least three different plants or three different leaves

To generate Cas9-induced mutations in *NtCPS2*, a vector was designed that harboured chimaeric guide RNA (gRNA) to guide Cas9 to target sequences where it bound and cleaved genomic DNA to generate double-strand breaks [22]. Two target sites of *CPS2* were selected (Supplementary Figure 1). The gRNA for each target site was generated by overlap-extension PCR. Cas9 was subcloned into a single expression vector [23]. The Cas9 and gRNA expression cassette was located in one expression vector (pRGEB32-Cas9-NPT II-*CPS2*-gRNA). Through the *Agrobacterium tumefaciens*-mediated method, 36 transformed regenerated plants in the T_0_ generation were obtained. After amplification with target-specific primers, all positive samples were sequenced to assess the mutation efficiency. Of 36 plants, eight were transgenic lines. Most of the transgenic lines had a single-base insertion of A, C, or T at Target 2. Thus, as the peptide chain was formed, the stop codon was encountered early in the process, and the translated amino acid chain was greatly shortened. To test the heritability of the mutations, homozygous transgenic plants in the T_0_, T_1_, and T_2_ generations were analysed. Detailed information about the homozygous T_2_ plants (M1–M9) is shown in Fig. 1A, and these plants were used for the following experiments.

### *NtCPS2* knockdown affects *cis*-abienol content

To verify whether the gene mutations caused changes in gene expression, quantitative real-time PCR (qRT-PCR) was used to detect the expression levels of *NtCPS2* in the leaves of mutant and wild-type (8306) plants. The results showed that *NtCPS2* expression decreased significantly in transgenic plants compared to wild-type plants (Fig. 1B). To detect changes in *cis*-abienol content in the leaves, exudates were collected from the mutant plants and analysed using GC-MS. The contents of *cis*-abienol also decreased significantly in mutant plants compared to wild-type plants (Fig. 1C). The results indicate that *NtCPS2* is one of the key genes regulating the *cis*-abienol biosynthesis pathway, and *NtCPS2* knockdown results in low levels of *cis*-abienol biosynthesis and accumulation. *NtABS* is another key gene involved in *cis*-abienol biosynthesis [16]. A previous study reported that *cis*-abienol was detected in plants expressing both *NtCPS2* and *NtABS* but not in plants expressing just one of the two genes [16]. *NtABS* expression was weak in the mutant plants compared to the wild-type plants, implying that *NtCPS2* knockdown negatively influenced *NtABS* expression. This is possibly because *NtCPS2* is located upstream of *NtABS* in the *cis*-abienol biosynthesis pathway.

### *NtCPS2* has a minor effect on the development of glandular trichomes in tobacco

**Fig. 2:**
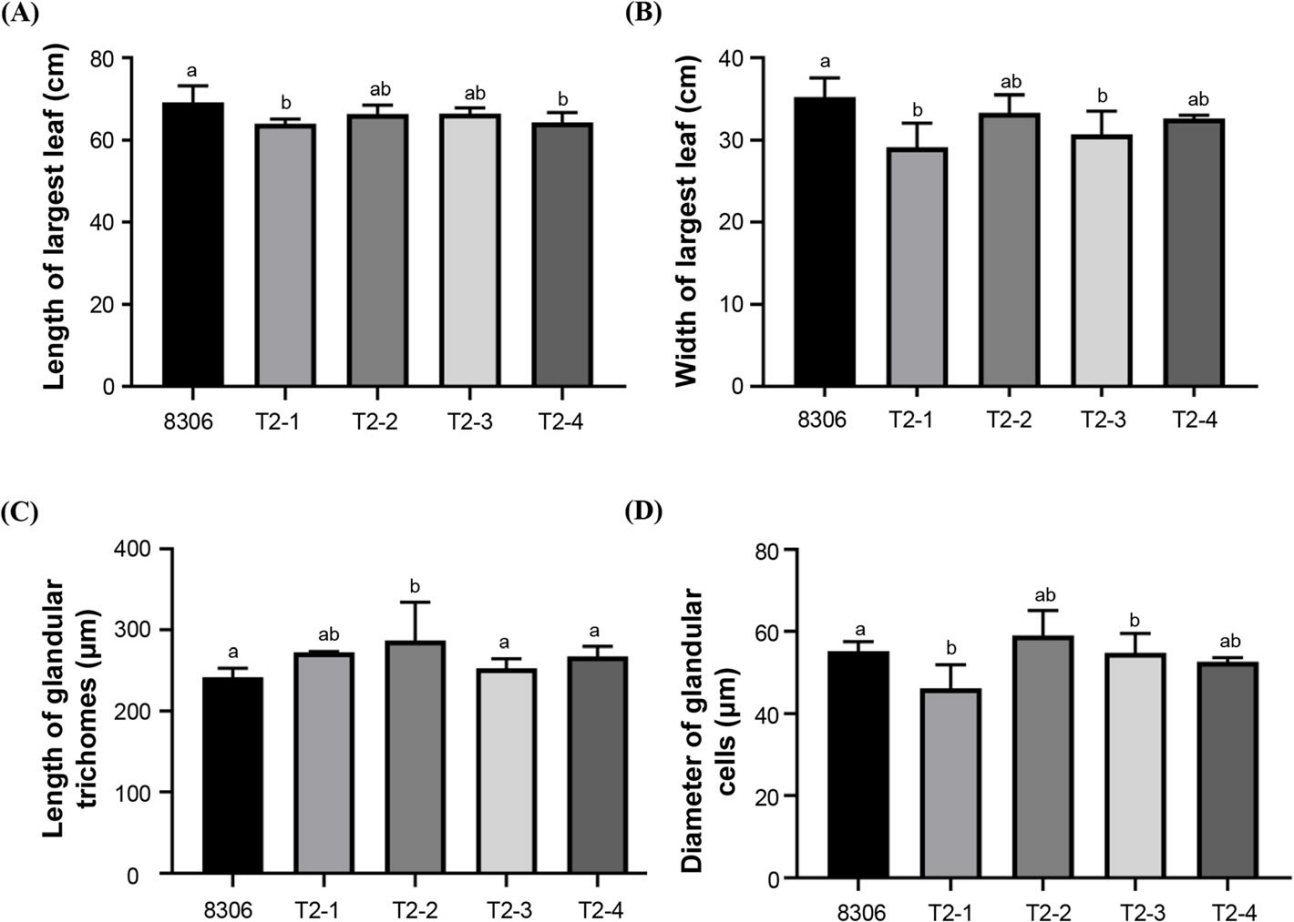
Morphological characteristics of mutant and wild-type plants, including the length **(A)** and width **(B)** of the largest leaf, length of glandular trichomes **(C)**, and diameter of glandular cells **(D)**. Values are presented as the means ± standard deviations (*n* = 4 for leaves and *n* = 100 for glandular trichomes). Different lowercase letters denote significant differences among plant lines (*p* < 0.05)

Agronomic characteristics were analysed to assess the mutant phenotypes (Fig. 2 and Supplementary Figure 2). Differences in plant height, internode length, number of leaves, and stem girth between mutant and wild-type plants did not exhibit the same trend. T2–2 mutants had longer internodes and wider stems than other mutants and wild-type plants, whereas all mutants except for T2–1 had shorter plant heights than the wild-type plants (Supplementary Figure 2). These results indicate that *NtCPS2* expression does not strongly affect tobacco plant morphology. As *NtCPS2* is specifically expressed in glandular cells [16], the morphology of the glandular trichomes on the largest leaf of each plant was examined. Both the length and width of the largest leaf were significantly shorter in mutant plants than in wild-type plants. The average diameter of glandular trichomes was smaller in mutant plants, especially T2–1, whereas both longer and shorter glandular trichomes were observed in mutant plants compared to wild-type plants (Fig. 2). Other trichome characteristics, such as the numbers of long and short trichomes, did not differ significantly between mutant and wild-type plants (data not shown). Thus, in the absence of *NtCPS2* expression in tobacco plants, the diameter of glandular cells and the area of the largest leaf decrease, but not the length of glandular trichomes. The T2–1 line was selected and used to profile transcriptomic changes after *NtCPS2* knockdown in tobacco 8306.

### Overview of transcriptome sequencing

To profile gene expression after *NtCPS2* knockdown, RNA-seq libraries were constructed for the mutant and wild-type plants. Six samples of each line were sequenced, and 41.64 G of clean data was obtained. In total, 6.70–7.02 G of effective data was collected from each sample, with a Q30 distribution of 94.29–94.91% and an average GC content of 43.41%. More than 95.58% of the clean reads had quality scores that met the Q30 criterion (probability of base-calling error = 0.1%) [24]. Furthermore, the GC content ranged from 43.15 to 43.66%. The sequencing data are summarized in Table 1.

**Table 1:** Summary of RNA-sequencing outcomes

Sample	Raw reads	Clean reads	Raw bases	Clean bases	Q30 (%)	GC (%)
Con1	49.05 M	47.90 M	7.36 G	6.89 G	94.60	43.46
Con2	49.76 M	48.74 M	7.46 G	7.02 G	94.88	43.25
Con3	47.66 M	46.51 M	7.15 G	6.70 G	94.29	43.30
L1	49.71 M	48.57 M	7.46 G	6.97 G	94.67	43.66
L2	49.77 M	48.72 M	7.47 G	6.99 G	94.88	43.41
L3	49.70 M	48.62 M	7.45 G	6.97 G	94.85	43.47

Not: Con, wild type; L, mutant

### Analysis of differentially expressed genes (DEGs) and their functions

**Fig. 3:**
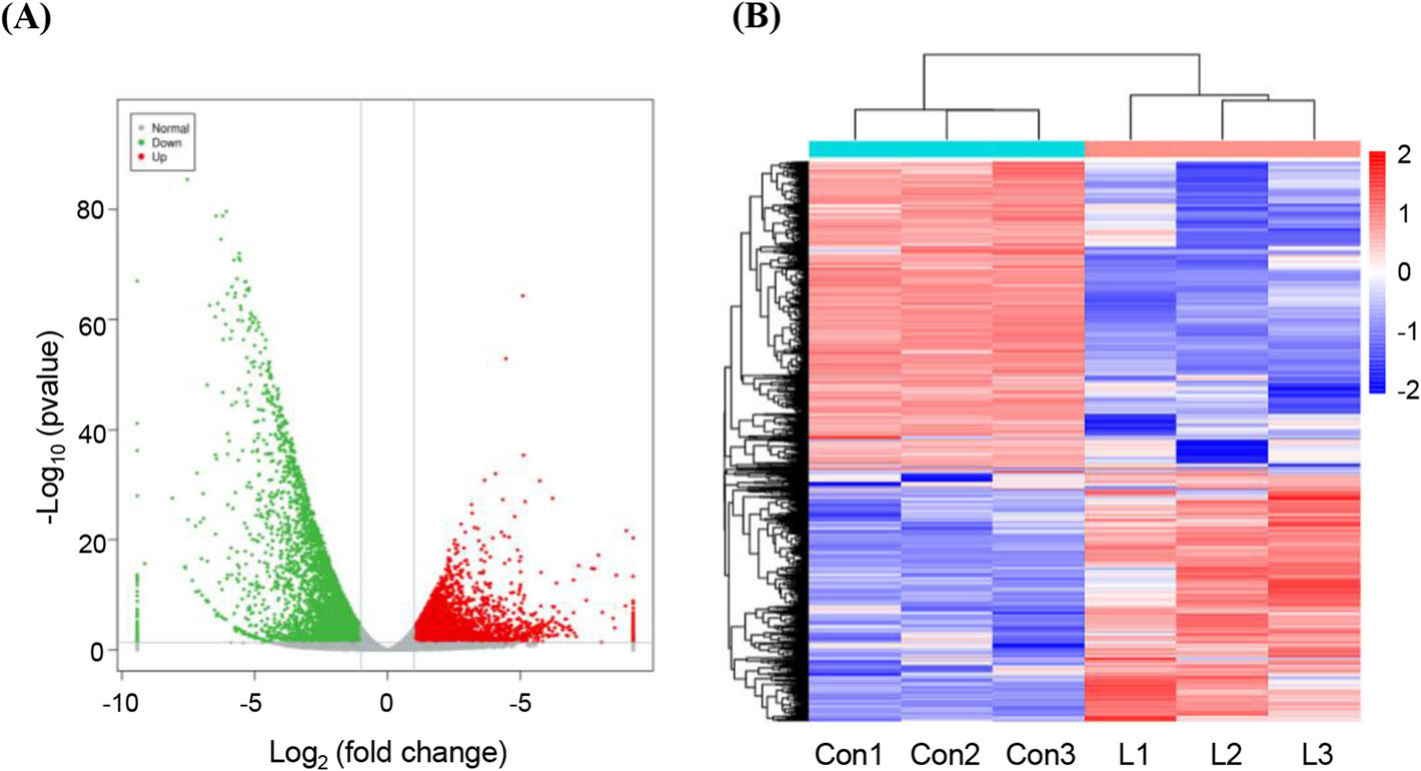
Differentially expressed genes (DEGs) were screened using an absolute log_2_(FC) value > 1 and *p*-value < 0.05. Significant differences in expression were observed for 9514 genes, as represented by a volcano plot **(A)** and heat map **(B)**

Volcano plots were used to assess the variation in gene expression between mutant and wild-type plants (Fig. 3A). In total, 9514 DEGs were detected. Among them, 4279 were upregulated and 5235 were downregulated in the transgenic tobacco plants compared to 8103 using the thresholds *p* < 0.05 and |log_2_(fold change [FC])| > 1 (Fig. 3B).

**Fig. 4:**
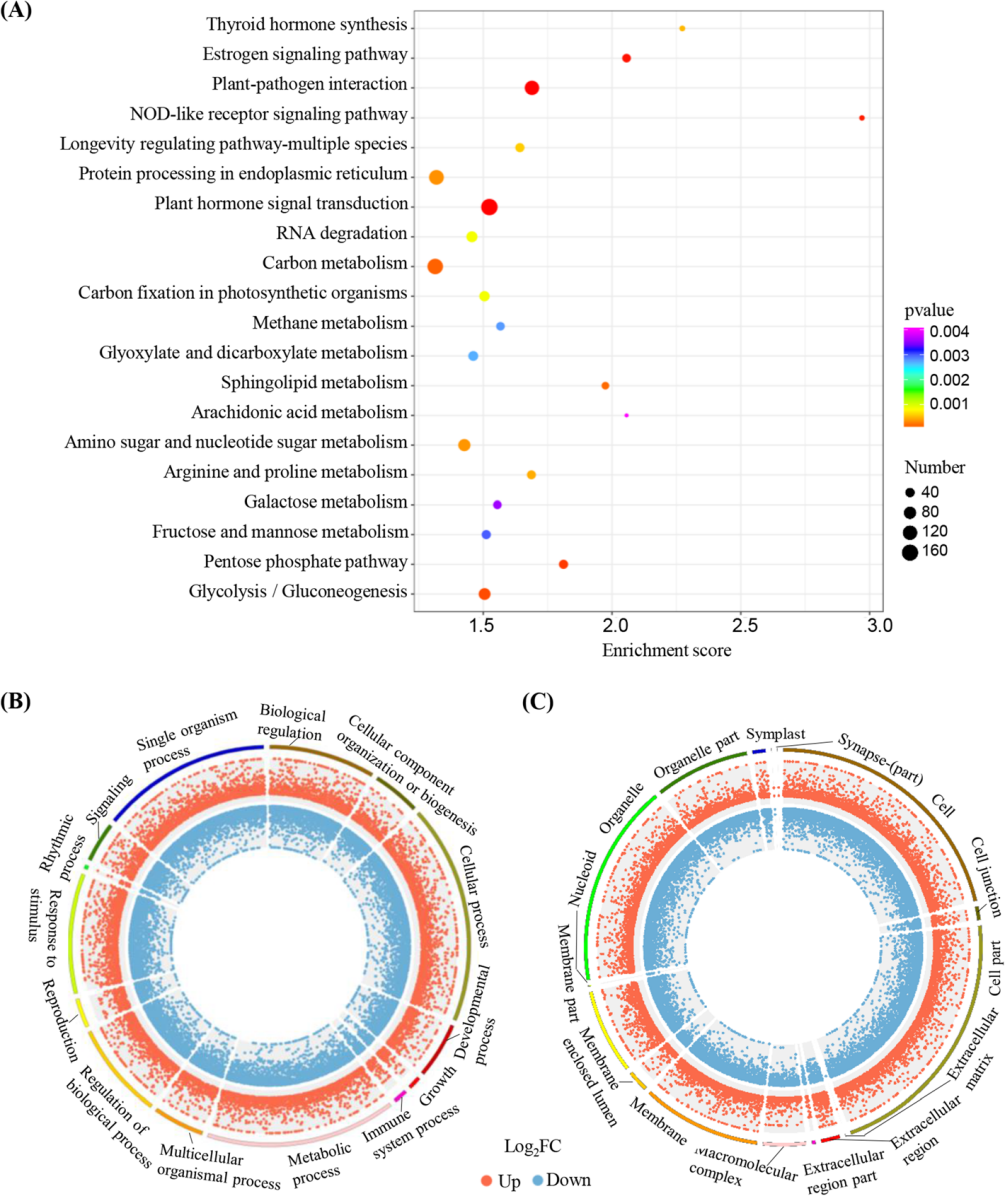
Kyoto Encyclopedia of Genes and Genomes (KEGG) and Gene Ontology (GO) pathway analyses of DEGs after *NtCPS2* knockdown. **A** The top 20 enrichment scores for KEGG pathway enrichment analysis of differentially expressed mRNAs. **B** GO annotations of DEGs assigned to the biological processes category. **C** GO annotations of DEGs assigned to the cellular components category

Kyoto Encyclopedia of Genes and Genomes (KEGG) and Gene Ontology (GO) pathway analyses of the differentially expressed mRNAs were performed to determine the functions of the DEGs. The 20 most significantly enriched pathways (lowest *q* values) according to KEGG metabolic pathway annotation were examined in detail (Fig. 4A). Based on GO analysis, the DEGs were most likely to be associated with biological processes (Fig. 4B) and cellular components (Fig. 4C). A large percentage of the DEGs were assigned to the categories metabolic process, cellular process, catalytic activity, binding, and single-organism process, with only a few genes assigned to channel regulator activity, cell killing, and protein tag. The DEGs involved in the pathways for diterpenoid biosynthesis, plant hormone signal transduction, and plant-pathogen interactions were analysed in detail.

### Validation of selected DEGs using qRT-PCR

**Fig. 5:**
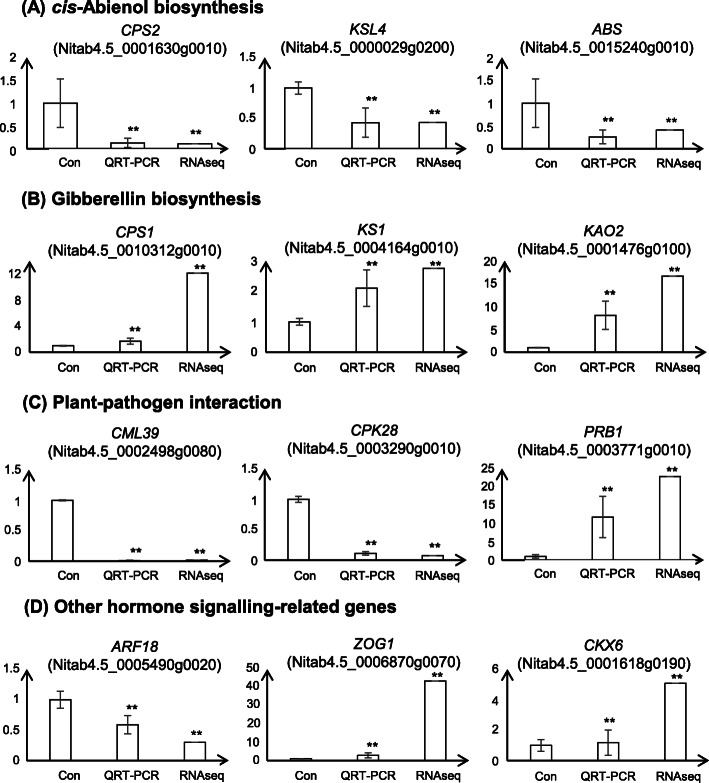
Transcription profiles of selected genes in mutant and wild-type plants as determined using RNA-seq and quantitative real-time polymerase chain reaction (qRT-PCR). Relative transcription levels of DEGs involved in *cis*-abienol biosynthesis **(A)**, gibberellin (GA) biosynthesis **(B)**, plant-pathogen interactions **(C)**, and other hormone-signalling pathways **(D)**. Asterisks indicate significant differences between samples and the control (*p* < 0.05, two-sample *t*-test)

To validate the RNA-seq data, 12 DEGs, including genes involved in *cis*-abienol and gibberellin (GA) biosynthesis as well as genes related to plant-pathogen interactions and other hormone signalling pathways, were selected randomly for qRT-PCR analysis. The gene expression patterns determined using qRT-PCR were consistent with those determined via transcriptome sequencing (Fig. 5). FC values differed between qRT-PCR and RNA-seq, possibly due to differences in the sensitivity of each method or because different samples were used for qRT-PCR and RNA-seq.

### Expression levels of genes related to *cis*-abienol biosynthesis decreased significantly in mutant plants

*NtCPS2* (Nitab4.5_0001630g0010) was identified as a DEG via RNA-seq, and its expression level was 9.27-fold lower in the mutant than in the wild type. The expression level of another key gene related to *cis*-abienol biosynthesis, *NtABS* (Nitab4.5_0015240g0010), also decreased 2.43-fold in the mutant. *NtCPS2* and *NtABS* operate in succession to synthesize *cis*-abienol [16]. When both *NtCPS2* and *NtABS* are expressed, *cis*-abienol is synthesized and can be detected in plants. However, *cis*-abienol synthesis does not occur in plants that express only one of these genes [16]. *NtCPS2* encodes 8-hydroxy-copalyl diphosphate synthase, which synthesizes 8-hydroxy-copalyl diphosphate, and *NtABS* encodes a kaurene synthase-like (KSL) protein, abienol synthase, which uses 8-hydroxy-copalyl diphosphate to produce *cis*-abienol. Our results indicate that weak expression of *NtCPS2* directly or indirectly results in a decrease in the expression level of *NtABS* and, consequently, low *cis*-abienol contents. Another putative *cis*-abienol synthase (Nitab4.5_0008024g0010) was also found to be downregulated in the mutant, indicating that this enzyme may have the same substrate as *NtABS* and thus be involved in the *cis*-abienol biosynthesis pathway. In contrast, other putative *cis*-abienol synthases, including Nitab4.5_0004164g0070 and Nitab4.5_0004164g0010, were found to be upregulated in the mutant. These two enzymes may have other functions in tobacco. Other DEGs involved in the *cis*-abienol biosynthesis pathway were also identified (based on KEGG analysis) and had lower expression levels in the mutant (Fig. 4). This included *KSL4* (Nitab4.5_0000029g0200, FC = 2.43) and genes predicted to encode ent-kaur-16-ene synthase (Nitab4.5_0002280g0060, FC = 5.61; and Nitab4.5_0002862g0030, FC = 1.57). As with *KSL4*, *NtABS* is a *KSL* gene (Table 2). Hence, *KSL4* and genes that putatively encode ent-kaur-16-ene synthase may be involved in *cis*-abienol biosynthesis. This needs to be verified in future work.

**Table 2:** Genes related to diterpenoid biosynthesis that are differentially expressed between *NtCPS2-*knockout and 8306 plants

Gene name	Gene ID	log_2_FC	Protein properties
*CPS2*	Nitab4.5_0001630g0010	−3.21	PREDICTED: copal-8-ol diphosphate hydratase, chloroplastic
*KSL4*	Nitab4.5_0000029g0200	−1.21	PREDICTED: ent-kaur-16-ene synthase, chloroplastic isoform X3
*DLO2*	Nitab4.5_0000129g0310	−2.53	PREDICTED: gibberellin 2-beta-dioxygenase 8-like
*GA2OX2*	Nitab4.5_0000222g0140	−5.09	PREDICTED: gibberellin 2-beta-dioxygenase 2
*GA2OX1*	Nitab4.5_0000923g0050	−3.82	PREDICTED: gibberellin 2-beta-dioxygenase 1-like
*GA2OX2*	Nitab4.5_0001013g0080	−2.88	PREDICTED: gibberellin 2-beta-dioxygenase 2-like
*KAO2*	Nitab4.5_0001476g0100	4.06	PREDICTED: ent-kaurenoic acid oxidase 1-like isoform X2
*GA20OX2*	Nitab4.5_0001573g0060	2.08	gibberellin 20 oxidase 1-like
*GA2OX1*	Nitab4.5_0002209g0240	−1.46	gibberellin 2-beta-dioxygenase 1-like
*KO*	Nitab4.5_0002280g0060	−2.49	PREDICTED: ent-kaurene oxidase, chloroplastic
*GA2*	Nitab4.5_0002862g0030	−1.19	PREDICTED: ent-kaur-16-ene synthase, chloroplastic-like isoform X1
*KS1*	Nitab4.5_0004164g0010	1.46	PREDICTED: *cis*-abienol synthase, chloroplastic-like
*TPS1*	Nitab4.5_0004164g0070	3.00	PREDICTED: *cis*-abienol synthase, chloroplastic-like
*GA2*	Nitab4.5_0008024g0010	−1.20	PREDICTED: *cis*-abienol synthase, chloroplastic-like
*CPS1*	Nitab4.5_0010312g0010	3.60	PREDICTED: ent-copalyl diphosphate synthase, chloroplastic-like isoform X1
*ABS*	Nitab4.5_0015240g0010	−1.28	*cis*-abienol synthase, chloroplastic

*FC* fold change

### GA biosynthesis increased significantly in mutant plants

**Fig. 6:**
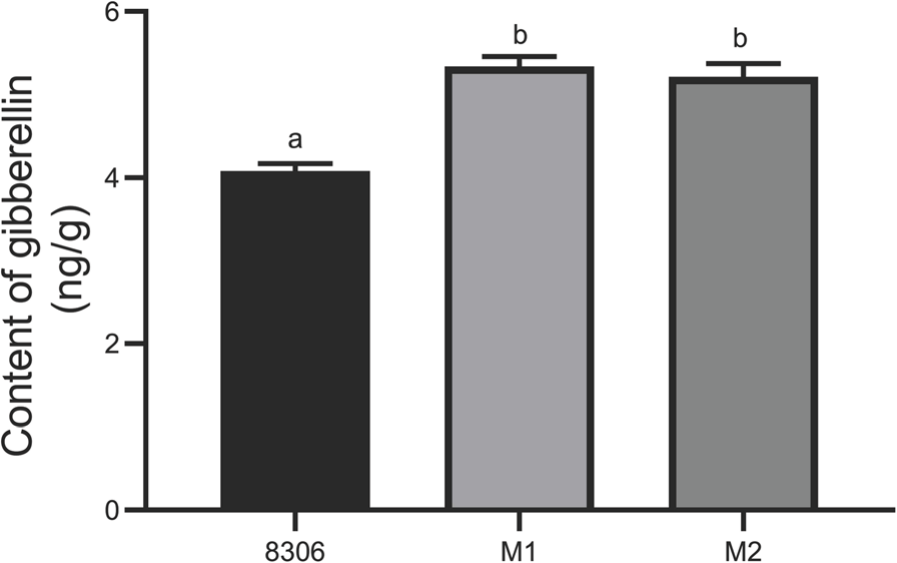
Gibberellin contents in the leaves of *NtCPS2-*knockdown (M1 and M2) and wild-type (8306) tobacco. Different lowercase letters denote significant differences among plant strains (*p* < 0.05)

According to diterpenoid biosynthesis pathways, the same substrate, geranylgeranyl pyrophosphate (GGPP), is used for *cis*-abienol and GA synthesis. In this study, most of the DEGs involved in GA biosynthesis were strongly upregulated in the mutant, including *KAO2* (Nitab4.5_0001476g0100, FC = 16.67), *KS1* (Nitab4.5_0004164g0010, FC = 2.76), and *CPS1* (Nitab4.5_0010312g0010, FC = 12.14) (Table 2). From these genes, ent-copalyl diphosphate synthase 1 (encoded by *CPS1*) and ent-kaurene synthase (encoded by *KS1*) were found to separately catalyse the synthesis of ent-kaurene from GGPP. However, *KO* (encodes ent-kaurene oxidase, which converts ent-kaurene to kaur-16-en-18-oate) expression was downregulated in the mutant. DEGs participating in the latter stages of the pathway, such as *KAO2* and *GA20*_*OX2*_, were upregulated compared to the wild-type plants. KO and KAO belong to the CYP701A, P450, and CYP88A clades. Accordingly, KAO is localized in the endoplasmic reticulum, whereas KO is localized in both the endoplasmic reticulum and plastid envelope [25]. The differential expression of *KO1* and *KAO2* in response to *NtCPS2* knockdown was explored further. GA contents in mutant plants were also analysed via GC-MS. The results showed that the GA contents in transgenic plants were significantly higher than those in wild-type plants (Fig. 6). GA12 is considered the precursor of all GAs in plants [26], and other GA forms are produced through oxidative steps catalysed by GA12. Genes involved in the production of these GA forms were up- and downregulated in the mutants.

### Changes in abscisic acid (ABA) biosynthesis and signal transduction in mutant plants

In carotenoid biosynthesis pathways, GGPP is also a substrate for ABA synthesis. RNA-seq analysis showed that four *PSY* genes (encoding phytoene synthases) were upregulated at the first step, which involves GGPP, in the mutant compared to the wild type. PSY is a transferase enzyme that is involved in the biosynthesis of carotenoids. It catalyses the conversion of GGPP to phytoene. Two genes encoding LCYs (lycopene epsilon cyclases) were also upregulated in the mutant at the next step. These results indicate that *NtCPS2* knockdown positively affects ABA synthesis, likely because substrate competition decreases. In addition, two ABA 8′-hydroxylases, which are involved in ABA degradation, were downregulated in the mutant. In the ABA signal transduction pathway, five of six ABA receptors (PYLs), which inhibit the expression of protein phosphatase 2C, were upregulated in the mutant. In the next step, serine/threonine-protein kinase expression was upregulated in the mutant. This might have been related to stress responses and stomatal opening and closure in tobacco leaves.

### Transcriptomic analysis of genes involved in plant-pathogen interactions

In plants, *cis*-abienol may participate in insect resistance and disease resistance [27]. Plant resistance to pathogen attack can induce the accumulation of pathogenesis-related proteins (PRs) that contribute to systematically acquired resistance. In this study, *PRs* were identified through RNA-seq. Of the 17 *PRs*, 14 (82.35%) were significantly upregulated in the mutant compared with the wild type, including genes that encode PR proteins 1A, B, and C (Table 3). Among the 17 families of PRs, PR 1–5, 9–11 and 17 were related to the acquisition of defence against pathogen infections. In addition, calcium is involved in regulating diverse physiological processes as a second messenger [28]. The results of transcriptomic analysis revealed that 15 of 19 CDPKs and most CAM/CML were significantly downregulated upon *NtCPS2* knockdown and a low content of *cis*-abienol, which disturbed the balance among active oxygen species, including rubidium hydroxide, reactive oxygen species, and nitric oxide synthase. Furthermore, the resistance of transgenic tobacco plants to black shank, induced by *Phytophthora nicotianae*, was checked. After the treatment of *P. nicotianae* for 7 days, wild-type plants showed wilting symptoms, while the transgenic tobacco plants were not (Supplementary Figure 3).

**Table 3:** Properties of DEGs encoding pathogenesis-related proteins

Gene name	Gene ID	log_2_FC	Protein properties
*PRB1*	Nitab4.5_0003771g0010	4.52	Pathogenesis-related protein 1A
*OSM34*	Nitab4.5_0004097g0050	3.76	PREDICTED: pathogenesis-related protein R minor form
*PRB1*	Nitab4.5_0014031g0010	3.51	PREDICTED: pathogenesis-related protein 1B-like
*–*	Nitab4.5_0006088g0020	3.46	PREDICTED: pathogenesis-related protein PR-4B
*–*	Nitab4.5_0018960g0010	3.35	PREDICTED: pathogenesis-related protein PR-4B
*–*	Nitab4.5_0008835g0020	3.27	PREDICTED: pathogenesis-related protein STH-2-like
*–*	Nitab4.5_0004861g0030	3.24	PREDICTED: pathogenesis-related protein 1C-like
*PRB1*	Nitab4.5_0004861g0040	3.16	PREDICTED: pathogenesis-related protein 1C
*–*	Nitab4.5_0008375g0050	3.06	PREDICTED: pathogenesis-related protein STH-2-like
*HEL*	Nitab4.5_0009495g0020	2.73	PREDICTED: pathogenesis-related protein PR-4A
*TL1*	Nitab4.5_0008011g0010	2.29	PREDICTED: pathogenesis-related protein 5-like isoform X1
*–*	Nitab4.5_0000194g0120	2.17	PREDICTED: pathogenesis-related protein STH-2-like
*CRF2*	Nitab4.5_0000105g0290	2.06	PREDICTED: pathogenesis-related genes transcriptional activator PTI6-like
*CRF2*	Nitab4.5_0002902g0060	1.11	PREDICTED: pathogenesis-related genes transcriptional activator PTI6-like
*MOS11*	Nitab4.5_0002073g0060	−1.47	PREDICTED: pathogenesis-related protein PRMS-like
*CRF2*	Nitab4.5_0000586g0010	−2.67	PREDICTED: pathogenesis-related genes transcriptional activator PTI6-like
*CRF2*	Nitab4.5_0007730g0010	−2.72	PREDICTED: pathogenesis-related genes transcriptional activator PTI6-like

## Discussion

**Fig. 7:**
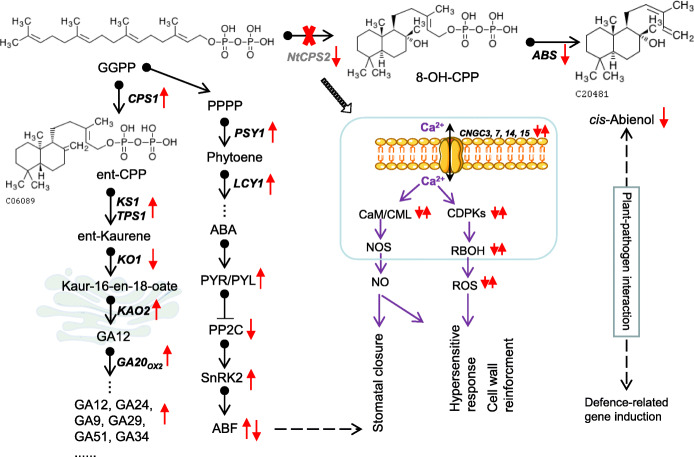
The changes in metabolic pathways associated with *NtCPS2* knockdown in tobacco mutants derived from KEGG database. 8-OH-CPP, 8-hydroxy-copalyl diphosphate; ABA, abscisic acid; ABS, *cis*-abienol synthase; CDPK, calcium-dependent protein kinase; CPS1, copalyl diphosphate synthase 1; GGPP, geranyl diphosphate; LCY1, lycopene epsilon cyclase 1; NO, nitric oxide; NOS, nitric oxide synthase; PP2C, protein phosphatase 2C; PPPP, prephytoene diphosphate; PSY1, phytoene synthase 1; RBOH, rubidium hydroxide; ROS, reactive oxygen species; SnRK2, serine/threonine-protein kinase 2

The aromatic characteristics of tobacco are improved by *cis*-abienol, which belongs to the labdane diterpenoid family. Although the genes encoding the enzymes participating in the two steps of *cis*-abienol biosynthesis have been cloned in tobacco [16], the function and transcriptome profile of *NtCPS2* knockdown are less well understood. By knocking down *NtCPS2*, whose product catalyses the first reaction in the *cis*-abienol biosynthesis pathway, we were able to examine how *cis*-abienol biosynthesis and other related metabolic pathways are controlled. The regulatory network is shown in Fig. 7.

### *NtCPS2* plays a limited role in the biosynthesis of *cis*-abienol and other terpenoids

Mutations in *NtCPS2* were previously reported to be strongly correlated with the absence of *cis*-abienol and labdene-diol in tobacco or a decrease in their levels [16]. In *N. sylvestris*, *cis*-abienol accumulates when both *NtCPS2* and *NtABS* are expressed [16]. In this study, we generated *NtCPS2-*knockdown tobacco lines using the CRISPR-Cas9 method. In mutant plants that weakly expressed *NtCPS2*, the levels of *cis*-abienol produced decreased (Fig. 1). *NtABS* is involved in the second step of *cis*-abienol biosynthesis, and its expression levels also decreased. The decreased expression of both of these genes might have resulted in low levels of the intermediate 8-hydroxy-copalyl diphosphate accumulating and the cessation of *cis*-abienol production downstream (Fig. 1C). The results indicate that *NtCPS2* plays a key role in *cis*-abienol biosynthesis; thus, downregulated gene expression leads to inactivation of the *cis*-abienol biosynthesis pathway.

The precursor GGPP, which participates in the first step of the pathway, is a common precursor for the biosynthesis of not only diterpenoids (including *cis*-abienol and labdene-diol) but also GA, carotenoids (including ABA), and the phytolchain of chlorophyll [29]. When NtCPS2 is absent, GGPP is not catalysed to produce 8-hydroxy-copalyl diphosphate, and other reactions that use GGPP as a substrate are enhanced. During GA biosynthesis in *Arabidopsis*, GGDP is converted to ent-kaurene in a two-step reaction catalysed by CPS and KS, which are encoded by *AtCPS* and *AtKS*, respectively [30, 31]. In this study, *NtCPS1* and *NtKS1* expression levels were upregulated after *NtCPS2* knockdown, and GA production, which occurs downstream, increased in the leaves of mutant plants (Fig. 6). In terms of carotenoid biosynthesis, genes (including *phytoene synthase 2* and *lycopene epsilon cyclase*) involved in converting GGPP to phytoene were upregulated after *NtCPS2* knockdown. Overall, reactions that consume GGPP as a substrate were enhanced. The results indicate that *NtCPS2* knockdown also contributes to the biosynthesis of other terpenoids depending on the same substrate. Future studies can verify this hypothesis by overexpressing *NtCPS2* and/or *NtABS.*

### Plants with mutations in *NtCPS2* still exhibit wild-type morphology

Diterpenoids such as cembranoid diterpenes and labdanoid diterpenes from tobacco-leaf exudates significantly influence cigarette smoke characteristics and flavour profiles [2, 3]. To our knowledge, no previous studies on the effects of *cis*-abienol on the growth and development of tobacco plants have been reported. We found that the mutant and wild-type morphology did not differ much, except for the diameter of the glandular trichomes. This indicates that *NtCPS2* knockdown and the subsequent decrease in *cis*-abienol do not affect tobacco plant morphology. However, the contents of other chemical substances (including GA and ABA) may change in mutants. Mutants had higher levels of GA and did not exhibit GA-overdose morphology. In *Arabidopsis*, CPS- and/or KS-overexpressing mutants also did not exhibit GA-overdose morphology [32]. This suggests that the levels of bioactive GA in these plants likely did not change. Transcriptomic analysis showed that the expression of GA20_OX2_ was upregulated, whereas that of GA2_OX4_, GA2_OX2_, and GA2_OX1_ was downregulated (Table 2). Wild-type *Arabidopsis* plants treated with exogenous GA and transgenic plants overexpressing the downstream GA biosynthesis gene *AtGA20*_*ox1*_ both exhibited aspects of GA overdose morphology [33]. The differential regulation of GA20_OX_, GA2_OX4_, GA2_OX2_, and GA2_OX1_ might result in different types of GA accumulating at different levels; thus, overall levels of bioactive GA may not change much, and plants may not exhibit a GA-underdose morphology.

### *cis*-abienol may participate in tobacco disease resistance

Labdanoid diterpenes may exhibit defence-related activities such as antifungal [9] and insecticidal [7, 8] activities [27, 34]. The application of *cis*-abienol to the roots of tobacco, tomato, and *Arabidopsis* at a concentration of 100 μmol/L can induce the expression of resistance genes and inhibit bacterial wilt disease [27]. In vitro experiments showed that concentrations of *cis*-abienol and related diterpenoids in the range of 0.01–100 ppm can inhibit the growth of *Phytophthora nicotianae* in tobacco [35]. However, *cis*-abienol isolated from *Cunninghamia konishii* had no inhibitory effect on the growth of wood decay fungi [36]. Kennedy et al. [37] found that the concentration of *cis*-abienol from 3.75 × 10^− 4^ μg/cm^2^ to 120 μg/cm^2^ had inhibitory effect on *Peronospora tabacina*. Compared with the control, the germination of sporangia was not affected by 10 kinds of *cis*-abienol concentrations. At the two lowest concentrations, 3.75 × 10^− 4^ μg/cm^2^ and 3.75 × 10^− 3^ μg/cm^2^, the incidence rate of *Peronospora tabacina* in tobacco was higher than that in the control group. In this study, the resistance of *NtCPS2* mutant tobacco lines to black shank was better than that of wild-type plants. Under field conditions, *cis*-abienol does not have an effect on diseased leaves in tobacco. QTL Phn15.1 in the cigar tobacco cultivar Beinhart 1000 was discovered, which provides a high level of partial resistance to black shank disease caused by *P. nicotianae* [13, 38]. A very close genetic association was found between Phn15.1 and the ability to biosynthesize *cis*-abienol. Recently, Steede et al. [35] observed no correlation between field resistance to *P. nicotianae* and the ability to accumulate *cis*-abienol in either transgenic materials or mapping populations. *Cis*-Abienol has little effect on black shank disease development under natural field conditions. Therefore, whether the accumulation of *cis*-abienol and the genes related to *cis*-abienol synthesis contributed to resistance against *P. nicotianae* in tobacco needs to be explored further. In this study, *NTCPS2* was edited to construct transgenic materials with low *cis*-abienol content. It was found that the transgenic materials were sick later than the wild-type in response to *Phytophthora nicotianae* treatment. In the subsequent experiments, we found that the ratio of GA_3_/ABA changed, which may have an impact on the resistance of transgenic materials.

A key defence response to pathogen attack in plants is the induction and accumulation of various PR proteins, which also contribute to systematically acquired resistance [39, 40]. The PR-1, PR-2 [41], PR-3, PR-4, PR-5 [42], PR-9 [43], PR-10 [44], PR-11 [45], and PR-17 [46] families are associated with acquired resistance to pathogen infections. Among the genes encoding these PR proteins, *PR-1* is generally considered a marker gene for disease resistance [47]. In this study, PR-related genes were both significantly up- and downregulated in the mutant plants (Table 3), implying that *cis*-abienol may participate in *Tobacco curly shoot virus* resistance in tobacco plants. Future research could assess disease resistance in *NtCPS2-*knockdown and *NtCPS2*-overexpressing mutants to clarify the contribution of *cis*-abienol to tobacco disease resistance.

## Conclusions

In this study, a genome-wide transcription profile was obtained for *NtCPS2-*knockdown tobacco plants edited using CRISPR-Cas9. *NtCPS2* is a key gene for *cis*-abienol biosynthesis in tobacco. Genes involved in the biosynthesis of *cis*-abienol, early metabolites of GA, and carotenoids (including ABA) were significantly differentially expressed after *NtCPS2* knockdown. The expression of PR-related genes also changed in response to low *cis*-abienol contents. Our findings may be useful for further investigation of the molecular mechanisms associated with *NtCPS2* gene function and the synthesis of *cis*-abienol. Additionally, our results can contribute to the development of high-aroma tobacco varieties.

## Methods

### Tobacco plant culture and inoculation

The tobacco plant variety (*N. tabacum* cv. 8306) used in this study produces high-aroma, flue-cured tobacco with high levels of *cis*-abienol. Plants were grown in scientific and educational park with loamy tidal soil of Henan Agricultural University, Zhengzhou City, China (113.63E, 37.75 N). Wild-type and transgenic tobacco plants were cultured and grown in mixed soil (1:1 vermiculite:humus) in a growth chamber at 22 °C with 250–300 μmol/m^2^/s photosynthetically available radiation and a 16-h light/8-h dark cycle. Measurements of leaf age started when the length of the middle leaf of each plant reached 1.5 cm. At a leaf age of 60 days, five tobacco plants at the same developmental stage from each group were selected, and the middle leaves were sampled for the measurements of morphological characteristics and RNA extraction. Seeds were collected at 25 days after flowering. *Phytophthora nicotianae* was cultivated at 24 °C on clarified V8-Agar, and Zoospores were produced under aseptic conditions [48]. The treatment was applied at the stage around third leaf stage of wild-type and transgenic tobacco plants. Small areas of source leaves were infiltrated with a suspension containing 600–900 zoospores μ/L for 7 days [49]. Control tissues were infiltrated with sterile tap water.

### Vector construction

Based on the mRNA sequences and corresponding genome sequences, two CRISPR target sites (Supplementary Table 1) were designed to improve gene-targeting efficiency. Target primers for PCR (Supplementary Table 2) were designed and synthesized. After primer synthesis, fragments containing the target sites were amplified using overlap-extension PCR. The amplified fragments were cloned into a CRISPR expression vector using a recombinant enzyme from Nanjing Novozan Biotechnology Co., Ltd. (Nanjing, China). The CRISPR vector was electroporated into *Escherichia coli*, and positive clones were screened using colony PCR for *Agrobacterium tumefaciens*-mediated transformation and tobacco gene transformation.

### *Agrobacterium*-mediated transformation

*Agrobacterium tumefaciens*-mediated transformation was performed as follows: 5 μL of recombinant plasmid was mixed with 50 μL of competent *Agrobacterium tumefaciens* cells on ice for 30 min. Blank YEB medium was added, and the mixture was incubated at 28 °C for 12–13 h. Then, the mixture was transferred to YEB solid medium containing 50 mg/L kanamycin and incubated at 28 °C for 36–48 h. Mature tobacco seeds were sterilized by washing with 75% alcohol and 10% sodium hypochlorite and placed into germination medium. The seeds were then grown under light for 45 days. Samples with a diameter of 0.5 cm were taken from leaves with a hole punch, transferred to a preculture medium, and incubated for 2 days under light. *Agrobacterium tumefaciens* was activated in medium containing 50 mg/L kanamycin. Leaf discs were infected with *Agrobacterium tumefaciens*, transferred to coculture medium, and left for 3 days. Thereafter, the leaf discs were washed with sterilized distilled water and antibiotics in an aqueous solution. After the leaves were dried, they were transferred to a screening medium and cultured under light. After differentiation, they were transferred to rooting medium. Transformed plants were obtained by rooting culture and transplanted into soil after 1 month.

### DNA extraction and sequencing for detecting mutations in the target gene

Leaflets were collected from each plant, and genomic DNA was extracted using a standard cetrimonium bromide protocol. NPTII-specific primers were used to detect successfully transformed plants via PCR (Supplementary Table 3). After confirming that the exogenous DNA fragment had been inserted, the primer 17KN48 was designed based on the *NtCPS2* gene sequence and target-site location to detect positive plants using PCR. PCR amplification was performed in the following reaction volume: 1 μL of DNA, 2 μL of 10 × PCR buffer, 0.4 μL of dNTP mixture, 0.2 μL of forward and reverse primers, 0.2 μL of rTaq DNA polymerase (TOYOBO, Osaka, Japan), and 20 μL of diethyl pyrocarbonate-treated water. PCR was carried out using the following programme: 94 °C for 3 min, 94 °C for 30 s, 55 °C for 30 s, 72 °C for 30 s, 72 °C for 10 min, and 25 °C for 1 min for 30 cycles. The PCR products were detected using gel electrophoresis and sequenced.

### Measurements of morphological characteristics of transgenic tobacco plants

Homozygous T_2_ tobacco plants were selected, and morphological parameters, including plant height, number of leaves, stem girth, internode length, and length and width of the largest leaf, were measured at a leaf age of 60 days. The morphology of leaf glandular trichomes was also characterized. The largest leaves of each plant of the same age were detached, and the epidermis at the centre of each leaf was peeled off to examine the glandular trichomes using an Axioplan 2 microscope (Carl Zeiss, Oberkochen, Germany). The numbers of long and short glandular trichomes were counted, and their lengths and diameters were measured. Each seedling had an average of approximately 100 glandular trichomes.

### Analysis of diterpenoids in leaf exudates using GC-MS

Leaf exudates were sampled from fresh tobacco leaves, and 1:1 portions of the samples were directly injected into a 6890 N gas chromatograph coupled to a 5973 N mass spectrometer (Agilent Technologies, Santa Clara, CA, USA) for GC-MS analysis. Tobacco diterpenoids were identified based on their mass spectra.

### RNA-seq analysis

The middle leaves of wild-type and transgenic tobacco were sampled at a leaf age of 60 days. Total RNA was extracted from frozen leaf samples using TRIzol reagent (Invitrogen, Carlsbad, CA, USA) according to the manufacturer’s protocol. RNA integrity was assessed using agarose gel electrophoresis, and RNA purity was checked using a NanoPhotometer® spectrophotometer (Implen, Munich, Germany). RNA concentration was quantified with a Qubit®2.0 Fluorometer using a Qubit® RNA Assay kit (Life Technologies, Carlsbad, CA, USA). From each qualified sample, 3 μg of RNA was sent to Illumina (San Diego, CA, USA) for sequencing. The cDNA library was prepared for sequencing according to the Illumina TruSeqTM RNA Sample Kit protocol. Sequencing was performed using an Illumina HiSeq 2500 system. RNA-seq reads were generated and processed to calculate expression levels, which were averaged over three biological replicates.

### Bioinformatics analysis of RNA-seq data

Raw reads were processed through in-house Perl scripts. Clean reads were obtained by removing adapter-containing reads, reads containing poly-N, and low-quality reads from the raw reads. The clean reads were then mapped to the tobacco reference genome (ftp://anonymous@ftp.solgenomics.net/genomes/Nicotiana_tabacum/assembly/K326). Using Hisat2 v2.0.5 (ftp://ftp.ensembl.org/pub/release-94/gtf/mus_musculus/), an index of the reference genome was built, and paired-end clean reads were aligned to the reference genome. We selected Hisat2 as the mapping tool because it can generate a database of splice junctions based on the gene model annotation file and thus produce better mapping results than other nonsplice mapping tools. The expression level of each gene was normalized to fragments per kilobase per million for comparison among different samples. Differential expression analysis was performed using the DESeq2 R package (1.16.1) [50], and an absolute log_2_(FC) value > 1 and a corrected *p*-value < 0.05 were set as the thresholds for DEGs for subsequent analysis.

DEGs were further annotated using GO functional enrichment analysis. GO terms with corrected *p*-values < 0.05 were considered to be significantly enriched for a given DEG. Clusters of orthologous groups and pathway analyses were performed using KEGG (http://www.genome.jp/kegg) analytical tools. We used the clusterProfiler R package [51] to test the statistical enrichment of KEGG pathways for the DEGs.

### Validation of DEGs using qRT-PCR

The differential expression of 20 genes between wild-type and transgenic tobacco leaf samples was confirmed using qRT-PCR analysis with three biological replicates per sample. Primer sets for the DEGs were designed using Primer Premier 5.0 (Premier Biosoft, San Francisco, CA, USA) and synthesised by Invitrogen Trading (Shanghai) Co., Ltd. (China). All primer sequences are listed in Supplementary Table 4. RNA isolation, cDNA synthesis, qRT-PCR, and statistical analyses were performed as previously described [52]. The expression levels of the DEGs were normalized to that of the internal control gene L25 [53].

### Statistical analyses

Data are presented as the means ± standard deviations. Two-sample t-tests were used to compare the means between two treatments. Comparisons across multiple treatments were performed using one-way analysis of variance followed by Tukey’s honestly significant difference post hoc test with SPSS v19 software (IBM Corporation, Armonk, NY, USA). A value of *p* < 0.05 was taken to denote statistical significance.

## Supplementary Information

**Additional file 1: Table S1.** The sequences of target sites.

**Additional file 2: Table S2.** The primers for PCR amplification.

**Additional file 3: Table S3.** NPTII specific primers and 17KN48 target primers.

**Additional file 4: Table S4.** Primer sequences for qRT-PCR..

**Additional file 5: Figure S1.** The target sites of *NtCPS2.*

**Additional file 6: Figure S2.** Morphological characteristics of mutant and wild-type plants, including plant height (A), internode length (B), number of leaves (C) and girth of stem (D). Values are presented as the means ± standard deviations (*n* = 4 for leaves and *n* = 100 for glandular trichomes). Different lowercase letters denote significant differences among plant lines (*p* < 0.05).

**Additional file 7: Figure S3.** Seedlings of wild-type and transgenic tobacco plants before (A) and after treatment of *Phytophthora nicotianae* infection for 7 days (B).

## Data Availability

The online version contains supplementary material available at https://www.ncbi.nlm.nih.gov/bioproject/?term=prjna734477, numbered PRJNA734477 in *Nicotiana tabacum* Raw sequence reads (TaxID: 4097).

## Abbreviations

CPS: Copalyl diphosphate synthase
ABA: Abscisic acid
GC-MS: Gas chromatography-mass spectrometry
ABS: Abienol synthase
RNA-seq: RNA sequencing
qRT-PCR: Quantitative real-time polymerase chain reaction
KSL: Kaurene synthase-like
KO: ent-kaurene oxidase
DEGs: Differentially expressed genes
KEGG: Kyoto encyclopedia of genes and genomes
GO: Gene ontology
PSY: Phytoene synthases
LCYs: Lycopene epsilon cyclases
PRs: Pathogenesis-related proteins
