# Supplementary figures and images for: RNA sequencing reveals transcriptomic changes in tobacco (Nicotiana tabacum) following NtCPS2 knockdown
Source: BMC Genomics. 2021 Jun 23;22:467. doi: 10.1186/s12864-021-07796-8 (PMC8220664; doi:10.1186/s12864-021-07796-8)

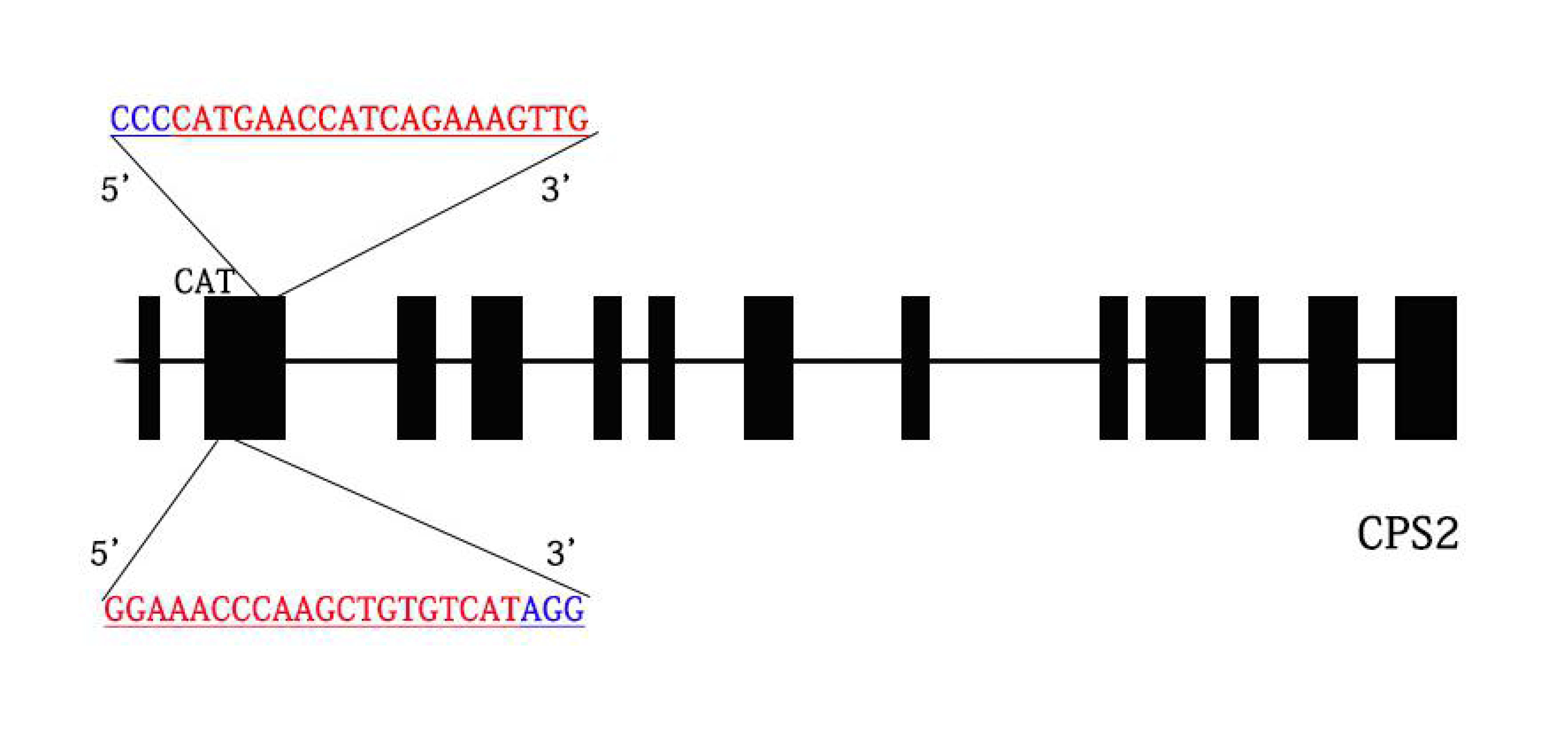

Supplement: Supplementary file 5 — Additional file 5: Figure S1. The target sites of NtCPS2. [file 12864_2021_7796_MOESM5_ESM.tiff]

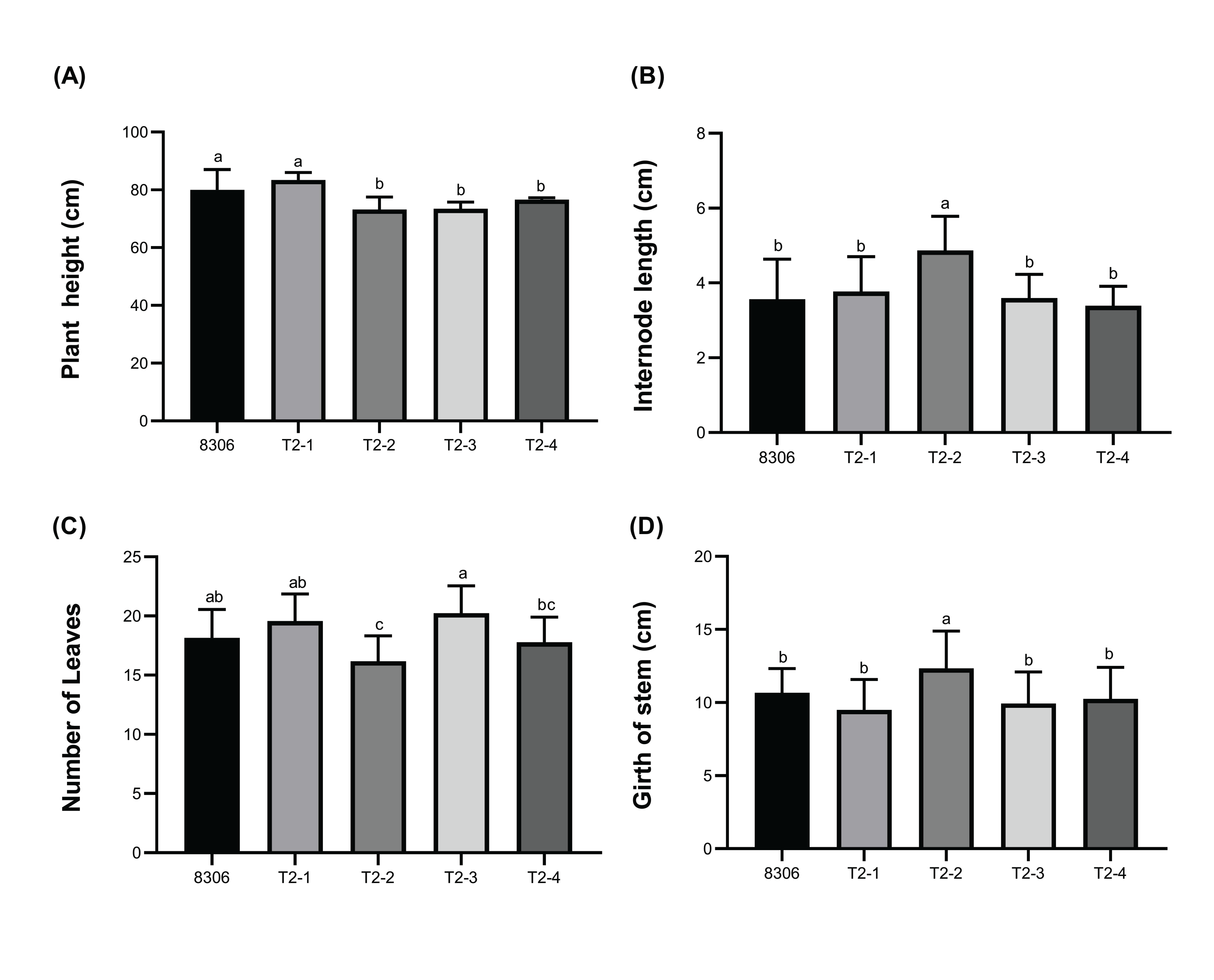

Supplement: Supplementary file 6 — Additional file 6: Figure S2. Morphological characteristics of mutant and wild-type plants, including plant height (A), internode length (B), number of leaves (C) and girth of stem (D). Values are presented as the means ± standard deviations (n = 4 for leaves and n = 100 for glandular trichomes). Different lowercase letters denote significant differences among plant lines (p < 0.05). [file 12864_2021_7796_MOESM6_ESM.tiff]

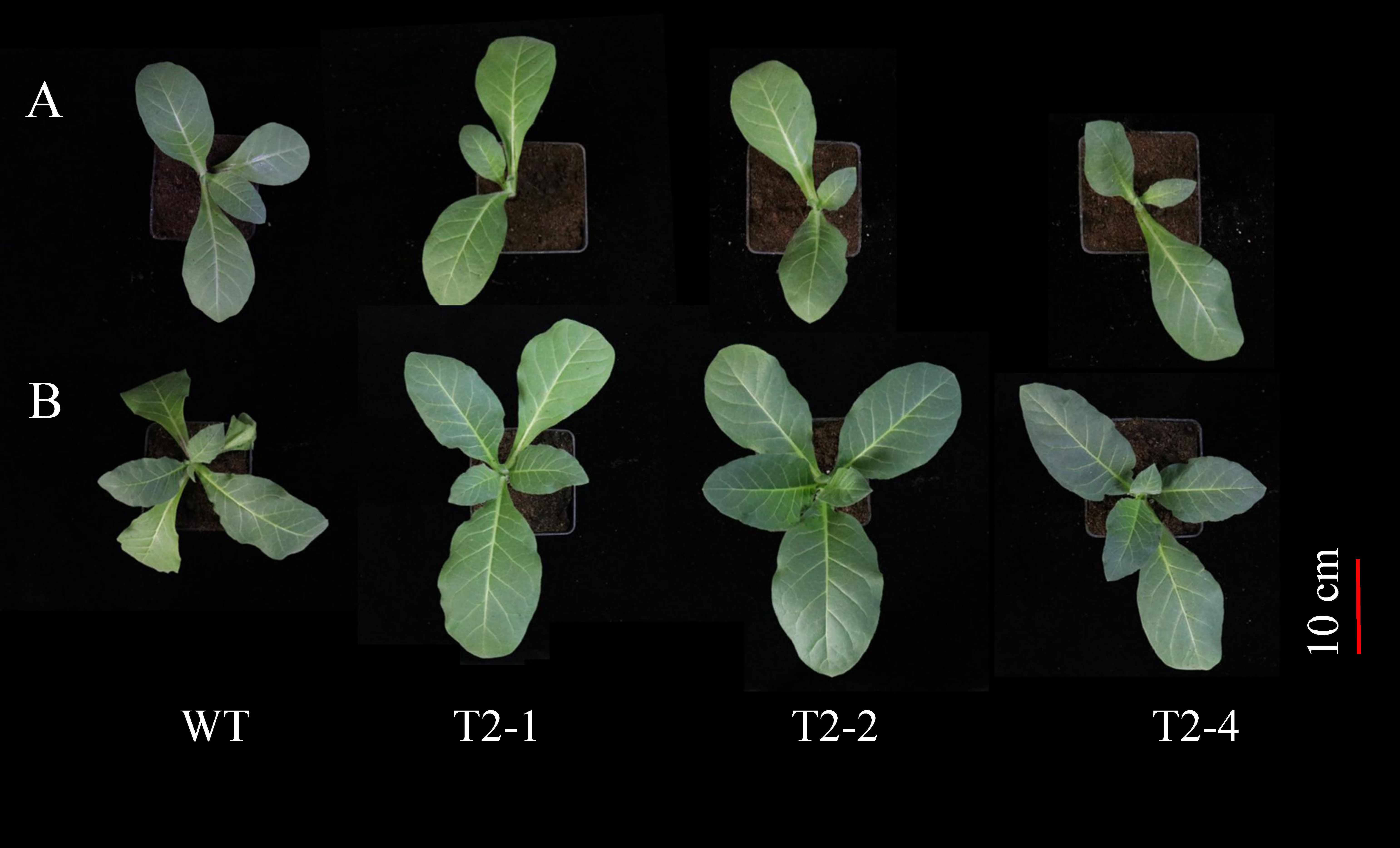

Supplement: Supplementary file 7 — Additional file 7: Figure S3. Seedlings of wild-type and transgenic tobacco plants before (A) and after treatment of Phytophthora nicotianae infection for 7 days (B). [file 12864_2021_7796_MOESM7_ESM.tiff]
